# The Occurrence of Tubercle Bacilli in the Faeces

**Published:** 1914-11

**Authors:** 


**The Occurrence of Tubercle Bacilli in the Faeces.** Laird, Kite, and Stewart (Journal of Experimental Medicine), give the following conclusions: Acid-fast bacilli have not often been found in the faeces of healthy persons; these have frequently been found in the stools of tuberculous individuals, and when present they are usually to be regarded as true tubercle bacilli. According to some authorities, bacilli are found in the faeces only in cases in which tubercle bacilli have been present also in the sputum. Others report their presence in the faeces not only when they have not been found in the sputum, but even when there has been no sputum to examine. Others, again, hold that the examination of the faeces should be of greater value than the examination of the sputum. The authors, after prolonged investigations, conclude that nearly all patients with tubercle bacilli in their sputum have also virulent tubercle bacilli in their faeces, as proved by animal inoculation; and further, that very few individuals whose sputum does not contain tubercle bacilli have acid-fast bacilli in their faeces.

